# What is infectiveness and how is it involved in infection and immunity?

**DOI:** 10.1186/s12865-015-0076-1

**Published:** 2015-03-26

**Authors:** Liise-anne Pirofski, Arturo Casadevall

**Affiliations:** Division of Infectious Diseases, Department of Medicine, Albert Einstein College of Medicine and Montefiore Medical Center, Room 610 Belfer Building, 1300 Morris Park Avenue, Bronx, NY 10461 USA; Department of Microbiology and Immunology, Albert Einstein College of Medicine, Bronx, NY USA

## Abstract

Proof of the Germ theory of disease and acceptance of Koch’s postulates in the late 1890’s launched the fields of microbial pathogenesis and infectious diseases and provided the conceptual framework that has guided thought and research in these fields. A central tenet that emerged from studies with microbes that fulfilled Koch’s postulates was that microbes that caused disease had characteristics that allowed them to do so, with the corollary that microbes that did not cause disease lacked disease-causing determinants. This observation, which held true for many diseases that were known to cause disease in the late 19^th^ century, such as toxin-producing and encapsulated bacteria, led to the view that the ability to cause disease rested with microbes and reflected the activity of specific determinants, or virulence factors. With the dawn of the 20^th^ century, efforts to neutralize virulence factors were under development and ultimately translated into anti-microbial therapy in the form of antibodies targeted to toxins and polysaccharide capsules. However, the 20^th^ century progressed, antibiotics were identified and developed as therapy for infectious diseases while other medical advances, such as specialized surgeries, intensive care units, intravenous catheters, and cytotoxic chemotherapy became commonplace in resourced nations. An unintended consequence of many of these advances was that they resulted in immune impairment. Similarly, HIV/AIDS, which emerged in the late 1970’s also produced profound immune impairment. Unexpectedly, the prevailing view that microbes were the sole perpetrators of virulence was untenable. Microbes that were rarely if ever associated with disease emerged as major causes of disease in people with impaired immunity. This phenomenon revealed that available explanations for microbial infectiveness and virulence were flawed. In this review, we discuss the question ‘what is infectiveness’ based on the tenets of the Damage-response framework.

## Introduction

The Germ theory was proven in the late 1890’s. For almost a century thereafter a question such as ‘what is infectiveness’ would have been considered naive. This is because after proof of Koch’s postulates and acceptance of the Germ theory, infectiveness was assumed to be a property of microbes that caused disease and microbes were considered to be solely responsible for disease pathogenesis with those that caused disease being fundamentally different than those that did not. These views gave rise to the concept that the ability to cause disease was a trait that stemmed from a particular microbial component, such as a virulence factor. This concept fit very well with microbial capsules, which could be identified with immune sera through capsular reactions, and toxins, which could be identified by toxicity in animals. An absence of these factors was considered to be sufficient for rendering a microbe non-pathogenic. The identification of virulence factors provided a rational basis for the development of pharmacological, genetic, and immunological ways to prevent their production and inhibit their modes of action. The latter resulted in the development of antibody-based therapies that mediated toxin neutralization and overcame the deleterious effects of capsular polysaccharides [1]. Antibody therapies that targeted virulence factors were the first rationally developed antimicrobial agents. Microbes with capsules and toxins were highly prevalent at the time the Germ Theory was developed and there were experimental platforms and animal models to probe their ability to cause disease. Although other microbes were also known to be able to cause disease, such as viruses, experimental tools to probe their pathogenicity were largely lacking. As such, there was no reason to question whether a microbe that was capable of causing disease would do so, or whether a microbe might cause disease in one host, but not in another. However, times change; and increasingly since the 1980s, a century after the Germ theory was proven, what is infectiveness has become a frequently asked question.

## Review

### What is infectiveness?

Infectiveness is best defined as the property of being infectious. Thus, infectiveness is part of infection. Infection is the event that occurs when a host acquires a microbe, or the microbe ‘infects’ the host [2]. Although the terms infection and disease are often erroneously used as synonyms, they are not synonyms as evidenced by the example that HIV infection is not the same as AIDS. For any given microbe and host at a given time in a given environmental context, infection results in an outcome in the host that is defined by microbial factors, host factors, and host-microbe interactions. For most microbes, these outcomes are: elimination, commensalism, colonization, disease, or latency. According to definitions put forth in the Damage-response framework, the states of commensalism, colonization, disease, and latency differ from one another by the amount of damage in the host [3]. The Damage-response framework, a theory of microbial pathogenesis, is discussed in detail in the following articles [2-6].

There are some clear read-outs of host damage, such as clinical signs and symptoms and laboratory and radiographic abnormalities. When damage reaches a certain threshold, there is clinical disease, though we note that clinicians and researchers would each benefit from the availability of more measures of host damage. Nonetheless, based on available tools, colonization, commensalism, disease and latency are associated with a degree of ‘evidence’ of host-microbe interaction. For commensalism, this evidence is presence of the microbiota. While for other states, this evidence is damage or inflammation in the host, which differs depending on the state. For latency, evidence of damage can vary but what is important is that the amount of damage is not manifested clinically. For *Mycobacterium tuberculosis*, the granuloma is a pauci-bacterial state that indicates the bacterium is present as well as a signature of host damage. This granuloma causes local damage in the lung but this damage does affect normal homeostasis, and consequently there is no disease. For herpesviruses, it is the presence of virus in rare cells that is indicative of the presence of virus in a host, whereas for hepatitis B and C, a latent state can be manifested by measureable virus without liver damage that is clinically manifest. In the latter instance, some might refer to the latent state as persistence. To the extent that latency and persistence each mean that the microbe is present in the host in a state that does not cause clinically manifest host damage, the terms are interchangeable.

As noted above, the Damage-response framework views the states of host-microbe interaction; commensalism, colonization, disease and latency, as continuous and different only in the extent of damage that occurs in the host. Hence, infection with a single microbe can result in more than one state in different hosts as well as in different states in a single host at different times, and sometimes simultaneously. For example, in a patient with bacteremia caused by *Staphylococcus aureus* who is colonized with this organism in the nares the same organism is present in the states of disease and colonization in the same individual. This highlights why, as put forth in the Damage-response framework [2,5], virulence cannot be a singular function of either microbe or host. In designating host damage as the read-out of host-microbe interaction, the Damage-response framework differs from theories of microbial pathogenesis that attribute virulence to either host or microbial factors. Therefore, infectiveness is defined by the quantity and quality of host damage that follows a host-microbe interaction. In this regard, infectiveness and virulence arguably represent the same property.

The word ‘infectiveness ’is etymologically related to the word infection. Given that infection is sometimes meant to convey contagiousness, the relationship of ‘contagiousness’ to ‘infectiveness’ must also be considered. Although infection is often associated with transmissibility, or communicability, only some microbes are transmitted from person. Modes of microbial transmission include person to person, vector to person, and environment to person. Although transmission, or communicability, is sometimes related to virulence, transmission and virulence are not necessarily linked. For example, *Candida albicans* is transmitted from mother to child soon after birth, but with the exception of occasional cases of neonatal candidiasis, acquisition of *Candida* at this time rarely causes damage or disease in immunologically intact individuals. Hence, transmission of *Candida* is generally not associated with virulence. On the other hand, viral agents, such as measles, are transmitted from person to person and transmission is generally associated with the development of disease in non-immune people. Thus, transmission of measles is associated with virulence, but only in a susceptible (non-immunized) host. In the case of *Cryptococcus neoformans*, the microbe is acquired from the environment. Infection is not always associated with disease and the nature of disease depends on the immune status of the host and the tissue that is infected. In the case of people with normal immunity, the microbe is limited to the lungs and the outcome is either elimination or a state of latency. On the other hand, in people with acquired immune impairment, most commonly due to HIV/AIDS or solid organ transplantation, but occasionally in people with what seems to be normal immunity, infection can transition to disease. This can happen by progression of an initial infection or when the fungus escapes the quiescent state of latency and disseminates from the lungs to the bloodstream and brain. These examples show that transmission is required for infection, and infection is required for damage and disease, but damage and disease depend on the immune status of the host in addition to microbial and environmental factors. Thus, ‘infectiveness’ is required for virulence, because infection is a necessary pre-condition for virulence, whereby virulence is the amount and degree of host damage stemming from host-microbe interaction. Time is also an important variable for virulence, because a microbe that does not cause damage in a host at one time might do so at another. For example, *Candida* can cause damage and disease long after infection originally occurred in someone who develops impaired immunity. On the other hand, measles might cause damage and disease in a non-immune person soon after infection. Thus, relationships between infectiveness and transmissibility and virulence are a complex function of time and microbial, host, and environmental factors.

For any given microbe, not all instances of infection result in the same outcome. This is because the degree and type of host damage is a function of characteristics of the microbe and the host response. The host response can vary from weak to strong, with weak and strong serving as basic terms to denote responses that are associated with a paucity of inflammation or an insufficient inflammatory response (weak) and those that are associated with too much inflammation or an excessive inflammatory response (strong). For most host-microbe interactions, neither weak nor strong responses are able to completely minimize host damage. Thus, either a weak or a strong host response can result in damage that translates into disease. In general, weak responses fail to restrict microbial growth and damage is primarily due to microbial burden. On the other hand, strong responses tend to produce damage due to excessive inflammation. The outcome of host-microbe interaction is also a crucial determinant of the response to antimicrobial therapy. For example, a weak host response is unlikely to be sufficient to induce microbial clearance, making antimicrobial chemotherapy necessary to reduce the microbial burden, with the caveat that drugs alone cannot always eradicate a microbe. On the other hand, although a strong host response (and/or effective antimicrobial therapy) might induce microbial clearance, host damage can occur due to ongoing inflammation. These scenarios each call for immune modulation. For example, patients with disseminated *Mycobacterium tuberculosis* infection can benefit from immunotherapy, such as adjunctive IFN-g therapy to enhance inflammatory responses, while individuals with tuberculous meningitis can benefit from corticorsteroids to reduce inflammation.

### The effect of host and microbial change on the outcome of host-microbe interaction

Progress in medicine from the early 1970’s onward brewed a perfect storm for infectious diseases and questions about microbial pathogenesis. This period witnessed the advent of powerful immunosuppressive drugs that transformed organ transplantation from a rare, high risk procedure into a standard, albeit specialized, operation [7], with the unintended consequence of inducing immune impairment. Similarly, other medical advances such as cytotoxic chemotherapy, radiation, and routine use of intravenous catheters also induced impaired cellular and mucosal immunity. This period also marked the onset of the HIV/AIDS pandemic, a global catastrophe [8] that rendered previously immunologically intact individuals profoundly immunodeficient. Together, HIV/AIDS, medical therapies, and interventions that caused immunosuppression led to the emergence of an unprecedented number of people with *de novo*, acquired impaired immunity [4]. These individuals were highly susceptible to the development of disease with certain microbes, some of which rarely if ever caused disease previously in immunocompetent people. These microbes included non-endemic fungi, most notably *Cryptococcus neoformans*, *Candida albicans*, and *Pneumocystis* spp*.* In addition, certain microbes exhibited an increased propensity to cause disease in patients with impaired immunity, including bacteria such as *Mycobacterium tuberculosis*, *Streptococcus pneumoniae*, *Haemophilus influenzae* Type B, *Neisseria meningiditis*, and bartonella; reactivated herpesviruses such as Epstein Barr virus, cytomegalovirus, and the etiologic agent of Kaposi’s sarcoma; and parasites such as *Toxoplasma gondii*. The emergence of diseases due to the foregoing microbes in immunocompromised patients led to intensive research that markedly advanced our understanding of host defense and the immune response to different microbes.

While the immune status of people changed, the ability of microbes to cause damage and disease also changed. This underscores the definition put forth in the Damage-response framework that virulence is the amount of damage that occurs in a susceptible host as a result of host-microbe interaction and that it cannot be defined independently of a host [2,5]. Beginning in the 1980’s, use, overuse, and abuse of broad-spectrum antibacterial drugs (antibiotics) led to the emergence of resistant bacteria, resulting in some strains that are resistant to all available antibiotics. Some antibiotic resistance is intrinsic, and some, such as that of *Staphylococcus aureus* and *Neisseria gonorrhea* to penicillin, began much earlier than the 1980’s. However, the crisis in antimicrobial therapy [9] became a catastrophe when it coincided with an expanding population of patients with impaired immunity and led to an inability to reliably treat infectious diseases in vulnerable patients. This failure of therapy was twofold. On one hand, there was a paucity of drugs to treat drug-resistant microbes. On the other hand, patients with impaired immunity were more susceptible to disease with microbes that rarely if ever caused disease in immunocompetent people, underscoring the central importance of the immune status of the host in microbial virulence.

Along with the emergence of resistant microbes, microbial niches changed too. For example, an increase in global travel led to the arrival of microbes with previously restricted borders in new venues, such as resistant *Streptococcus pneumoniae* (pneumococcus), which spread from Spain to the rest of the world [10,11]. Pneumococcus and many other bacteria need to travel from person to person, but microbes that do not require human hosts were also on the move. West Nile virus, a mosquito borne microbe (arbovirus), arrived in the northeastern United States with a change in avian migration [12]. In addition, climate change, particularly global warming and other weather events such as El Nino, tornados, and other catastrophes, led to epidemics of cholera and other water borne microbes [13]. Ecological changes, climate change, and changes in sanitation and water handling promote vector migration and vector borne zoonoses [14]. An increase in fungal diseases in mammalian hosts has been hypothesized to follow global warming [15]. Hence, environmental forces have altered microbial niches and fostered microbial proliferation.

### The involvement of innate immunity in infectiveness

A major discovery that changed our understanding of the relationship between immunity and infectiveness was the discovery of pattern recognition receptors (PRRs) and the subsequent unraveling of their role in host defense [16,17]. PRRs include toll like receptors (TLRs) and other, mainly C-type lectin, receptors on host immune cells and tissues that recognize microbial determinants. The discovery of these moieties identified the initial interaction between a microbe and immune cell or tissue as a critical determinant of the success or failure of host defense, underscoring the pivotal role of the host-microbe relationship in the development of immunity. Our understanding of PRR-microbial interaction was initially limited to a few examples, such as TLR2 binding to microbial cell wall glucans, lipoteichoic acid, and peptidoglycans; TLR4 binding to lipopolysaccharide and HSP60, TLR5 binding to flagellin; and TLR9 binding to CPGs. Subsequently, C-type lectin PRRs, such as dectin-1, which bind fungal cell wall determinants, retinoic acid inducible gene-like receptors (RLRs) that bind viral RNAs, such as RIG-1, were identified as were the signaling pathways that result in the production of inflammatory cytokines and mediators, such as those mediated by nucleotide-binding domain, leucine-rich repeat- containing (NOD) proteins [18]. Together the discovery of PRRs and their signaling pathways revealed critical mechanisms by which host-microbe interaction at the host cell interface leads to microbial clearance and immunity. Evasion of PRR binding is a mechanism by which some microbes can avoid or subvert host defense. There is now abundant evidence that genetic polymorphisms and defects in PRRs and their downstream signaling and cytokine production pathways predispose certain patients to disease with certain microbes [19]. Thus, genetic defects and the infectious diseases to which they predispose patients can serve as sentinels that define the relationship between immunity and infectiveness. Such defects include those that affect the response to many microbes, via global pathways, as well as those that affect the response to specific microbes [20,21]. The complex role of genetics in susceptibility to disease is highlighted by the way in which mutations affect immune responses to Epstein Barr virus infection [22].

Another recently recognized component of the innate immune system that links immunity to infectiveness is the IgM memory B cell repertoire [23]. Circulating and marginal zone IgM memory B cells with the phenotype IgM^hi^IgD^lo^CD27^+^ are now recognized as ‘first responders’ in the host response to microbes. People who lack these cells, which are depleted in patients with HIV infection (see [24]), aging [25], and common variable immunodeficiency [26] are more susceptible to diseases caused by encapsulated microbes such as *Streptococcus pneumoniae* [27] and *Cryptococcus neoformans* [24]. The origin of these cells and the extent to which they are homologs of mouse B1-B cells remains a matter of debate. Nonetheless, they have been linked to serological memory for microbes for over a decade [28]. IgM memory B cells are known to be activated in a T-independent fashion in the absence of antigen stimulation, including by CPGs via TLR9, and to produce natural IgM that binds conserved microbial determinants, such as cell wall carbohydrates [23,29,30].

### The involvement of infectiveness in acquired immunity

The ability of microbial determinants to induce immunity and protection against infectious diseases has been recognized for more than a century. Since the development of immune sera as the inaugural antimicrobial agents, the power of induced immunity to defined microbial determinants has been harnessed in vaccines. In fact, resourced countries where infant/childhood vaccination is standard have enjoyed an unprecedented sense of safety from childhood diseases due to vaccine-mediated protection against measles, rubella, mumps, varicella, polio, pertussis, *Haemophilus influenzae* Type B, and pneumococcus. At the present time, even where there is access to vaccines, vaccine refusal, serotype replacement, and uncertainty about the duration of protection pose roadblocks to complete prevention of pertussis, mumps, measles, and pneumococcus. In addition, currently there are no licensed vaccines for malaria or other parasites, tuberculosis, dengue, fungi, or drug resistant bacteria. Nonetheless, immunization has markedly reduced rates of the aforementioned diseases, eliminated smallpox globally, and provides hope that other vaccine-preventable diseases can be eliminated. The ability of immunization to prevent infectious diseases provides incontrovertible evidence of the essential role of host immunity in disease prevention. Immune people rarely develop full-blown disease. Vaccine-elicited protection is ‘ready-made’ as long as the vaccine recipient has had enough time to develop immunity. On the other hand, natural immunity can take time to develop in a naïve host and is not always sufficient to prevent disease. As for the effect of acquired immunity on infectiveness, immunity prevents disease, but might not prevent infectiveness. Some vaccines do not prevent infection despite their ability to prevent disease and some vaccines, measles, mumps, rubella, and varicella are live agents.

## Summary and conclusions

In this review, we have addressed the question ‘what is infectiveness’ based on definitions of microbial pathogenesis and virulence put forth in the Damage-response framework. Our take home message is that ‘infectiveness’, like virulence and the states in which microbes are found in hosts, is an outcome of host-microbe interaction that is a complex function of time and microbial, host, and environmental factors independently and in combination. However, the emergence of new infectious diseases and occurrence of epidemics challenge the ability of available theories and paradigms to incorporate new information. Along these lines, the seemingly infinite combinations of host, microbe, and environment that can emerge in any given host-microbe interaction begs another question: how can new diseases and discoveries about hosts and microbes be incorporated into current thinking? Here is our answer: we think the following aspects of the Damage-response framework [4] provide a way to address this challenge:

1. The only terms that are required to explain microbial pathogenesis are ‘host’ and ‘microbe’ with the relevant read-out of their interaction being damage in the host; thus, designations of microbes as ‘pathogens’, ‘non-pathogens’, ‘primary pathogens’, ‘commensals’ are irrelevant.
  - Host damage can be a function of host or microbial properties, or both.
2. Four states represent the outcome of host-microbe interaction: commensalism, colonization, disease, and latency.
  - The states are considered to be continuous and to differ only in the degree of damage in the host as a function of time.
3. Virulence is a microbial property that is defined by the outcome of host-microbe interaction.
  - Virulence is an emergent property; it cannot be predicted by host or microbial properties alone [31].
  - As an emergent property, virulence is influenced by factors that affect microbial and host fitness, including behavioral, environmental, political, as well as host and microbial factors.
  - Thus, the Damage-response framework is able to account for differences in the outcome of infection between hosts (i.e. the same microbe can cause damage and disease in one host, but not another) and within hosts (i.e. the same microbe can cause damage and disease at one time, but not after immunization, prior infection, or under another set of conditions).

## References

[1] Casadevall A, Scharff MD (1995). Return to the past: the case for antibody-based therapies in infectious diseases. Clin Infect Dis.

[2] Casadevall A, Pirofski L (1999). Host-pathogen interactions: redefining the basic concepts of virulence and pathogenicity. Infect Immun.

[3] Casadevall A, Pirofski L (2000). Host-pathogen interactions. II. The basic concepts of microbial commensalism, colonization, infection and disease. Infect Immun.

[4] Casadevall A, Pirofski L (2003). The damage-response framework of microbial pathogenesis. Nat Rev Microbiol.

[5] Casadevall A, Pirofski L (2001). Host-pathogen interactions: the attributes of virulence. J Infect Dis.

[6] Casadevall A, Pirofski L. What is a host? Incorporating the microbiota into the damage-response framework. Infect Immun. 2014.In press. 10.1128/IAI.02627-14 PMC4288903 25385796

[7] Linden PK (2009). History of solid organ transplantation and organ donation. Crit Care Clin.

[8] De Cock KM, Jaffe HW, Curran JW (2011). Reflections on 30 years of AIDS. Emerg Infect Dis.

[9] Casadevall A (1996). Crisis in infectious diseases: time for a new paradigm?. Clin Infect Dis.

[10] Harbarth S, Samore MH (2005). Antimicrobial resistance determinants and future control. Emerg Infect Dis.

[11] Pang T, Guindon GE (2004). Globalization and risks to health. EMBO Rep.

[12] Rappole JH, Derrickson SR, Hubalek Z (2000). Migratory birds and spread of West Nile virus in the Western Hemisphere. Emerg Infect Dis.

[13] Altizer S, Ostfeld RS, Johnson PT, Kutz S, Harvell CD (2013). Climate change and infectious diseases: from evidence to a predictive framework. Science.

[14] Altizer S, Bartel R, Han BA (2011). Animal migration and infectious disease risk. Science.

[15] Garcia-Solache MA, Casadevall A. Global warming will bring new fungal diseases for mammals. MBio. 2010;1(1). 10.1128/mBio.00061-10 PMC2912667 20689745

[16] Akira S, Uematsu S, Takeuchi O (2006). Pathogen recognition and innate immunity. Cell.

[17] Broz P, Monack DM (2013). Newly described pattern recognition receptors team up against intracellular pathogens. Nat Rev Immunol.

[18] Ting JP, Duncan JA, Lei Y (2010). How the noninflammasome NLRs function in the innate immune system. Science.

[19] Netea MG, van de Veerdonk FL, van der Meer JW (2012). Primary immunodeficiencies of pattern recognition receptors. J Intern Med.

[20] Alcais A, Abel L, Casanova JL (2009). Human genetics of infectious diseases: between proof of principle and paradigm. J Clin Invest.

[21] Picard C, Abel L, Casanova JL (2007). Human monogenic disorders that confer predisposition to specific infections. Novartis Found Symp.

[22] Bassiri H, Janice Yeo WC, Rothman J, Koretzky GA, Nichols KE (2008). X-linked Lymphoproliferative Disease (XLP): a model of impaired anti-viral, anti-tumor and humoral immune responses. Immunol Res.

[23] Carsetti R, Rosado MM, Wardmann H (2004). Peripheral development of B cells in mouse and man. Immunol Rev.

[24] Subramaniam K, Metzger B, Hanau LH (2009). IgM(+) memory B cell expression predicts HIV-associated cryptococcosis status. J Infect Dis.

[25] Shi Y, Yamazaki T, Okubo Y, Uehara Y, Sugane K, Agematsu K (2005). Regulation of aged humoral immune defense against pneumococcal bacteria by IgM memory B cell. J Immunol.

[26] Agematsu K, Futatani T, Hokibara S (2002). Absence of memory B cells in patients with common variable immunodeficiency. Clin Immunol.

[27] Kruetzmann S, Rosado MM, Weber H (2003). Human immunoglobulin M memory B cells controlling Streptococcus pneumoniae infections are generated in the spleen. J Exp Med.

[28] Bernasconi NL, Taggiani E, Lanzavecchia A (2003). Maintenance of serological memory by polyclonal activation of human memory B cells. Science.

[29] Capolunghi F, Rosado MM, Sinibaldi M, Aranburu A, Carsetti R (2013). Why do we need IgM memory B cells?. Immunol Lett.

[30] Lanzavecchia A, Sallusto F (2009). Human B cell memory. Curr Opin Immunol.

[31] Casadevall A, Fang FC, Pirofski LA (2011). Microbial virulence as an emergent property: consequences and opportunities. PLoS Pathog.

